# The Role of TCA Cycle Anaplerosis in Ketosis and Fatty Liver in Periparturient Dairy Cows

**DOI:** 10.3390/ani5030384

**Published:** 2015-08-18

**Authors:** Heather M. White

**Affiliations:** Department of Dairy Science, University of Wisconsin-Madison, Madison, WI 53706, USA; E-Mail: heather.white@wisc.edu; Tel.: +1-608-263-7786; Fax: +1-608-263-9412

**Keywords:** periparturient dairy cow, fatty liver, ketosis, anaplerosis, TCA cycle, fatty acid oxidation

## Abstract

The transition to lactation period in dairy cattle is characterized by metabolic challenges, negative energy balance, and adipose tissue mobilization. Metabolism of mobilized adipose tissue is part of the adaptive response to negative energy balance in dairy cattle; however, the capacity of the liver to completely oxidize nonesterified fatty acids may be limited and is reflective of oxaloacetate pool, the carbon carrier of the tricarboxylic acid cycle. Alternative metabolic fates of acetyl-CoA from nonesterified fatty acids include esterification to triacylglycerides and ketogenesis, and when excessive, these pathways lead to fatty liver and ketosis. Examination of the anaplerotic and cataplerotic pull of oxaloacetate by the tricarboxylic acid cycle and gluconeogenesis may provide insight into the balance of oxidation and esterification of acetyl-CoA within the liver of periparturient dairy cows.

## 1. Introduction

The transition to lactation period is the most metabolically challenging period for dairy cattle and is characterized by homeorhetic changes, negative energy balance (NEB), and increased risk for metabolic and reproductive disorders. Over 60% of all dairy cows develop fatty liver during the transition to lactation period, which is a direct response to an energy imbalance, and may decrease hepatic efficiency, animal health, and milk production. Recent research suggests that regulation of key hepatic genes may play an important role in liver lipid accumulation and ketone accumulation during this period.

## 2. Ketosis and Fatty Liver Disease

Voluntary intake reduction around the time of calving, coupled with increases in energy requirements to meet the needs of lactation, results in cows entering a state of NEB. During periods of NEB, triglycerides (TAG) are mobilized from adipose stores [1,2,3,4,5,6,7]. At the adipose tissue, activation of hormone-sensitive lipase (HSL) through the protein kinase A cascade results in phosphorylated HSL translocation to the lipid droplet and hydrolyzing TAG to non-esterified fatty acids (NEFA) and glycerol [8,9]. In addition to HSL, phosphorylation of perilipin plays a key role in lipolysis during the transition to lactation period [8]. Conversely, adipose triglyceride lipase, which is important in lipolysis in other species, is downregulated during the transition to lactation in dairy cows [8]. 

Once liberated, NEFA can be metabolized by many maternal and fetal tissues for energy production and fat synthesis [10,11,12]. Circulating NEFA is a significant contributor to milk fat synthesis in the mammary gland postpartum and can contribute to almost half of milk fat [12,13]. During neutral or positive energy balance, when plasma NEFA are not elevated, NEFA contributes only minimally to milk fat synthesis [12,13]. Nonesterified fatty acids are also largely metabolized by hepatic tissue. Uptake of NEFA from plasma by the liver is proportional to the plasma concentration and the rate of blood flow and is about 25% of NEFA passing through hepatic circulation [14,15]. During the transition to lactation and associated surge in plasma NEFA, rate of blood flow increases [16]. This combination results in an increase uptake of NEFA into hepatic tissue in postpartum dairy cows [7,15].

Once taken up by the liver, fatty acids are β-oxidized to acetyl-CoA units with four possible fates: complete oxidation through the TCA cycle, incomplete oxidation through ketogenesis, TAG synthesis and packaging as very-low density lipoprotein for export from the liver (minimal in ruminant animals), or TAG synthesis for storage as liver lipids [3]. When available acetyl-CoA exceeds the capacity of the TCA cycle, there is increased production of ketone bodies and deposition of TAG, leading to the onset of ketosis and fatty liver syndrome. While circulating ketone bodies can be used to a certain extent as a fuel source by heart, brain, liver, and mammary tissue, excessive blood ketone bodies can have negative effects on animal health and productivity. Hyperketonemia, the presence of elevated circulating ketone bodies, is defined as blood BHBA concentration ≥3.0 mM [17]. Sub-clinical ketosis (SCK), hyperketonemia without clinical symptoms of ketosis, is diagnosed when blood BHBA concentration ≥1.2 mM. Incidence of SCK is between 15% to 60% of dairy cows [17,18] while clinical ketosis occurs in 2% to 15% of cows [18]. 

Often developing in parallel, accumulation of liver lipids during early lactation can be as high as 500 g/d, and it is predicted that 60% of dairy cows have severe or clinical fatty liver, defined as a liver lipid content greater than 10% on wet weight basis at one day postpartum [4,19]. Accumulation of liver lipids is a result of a combination of increased fatty acids delivered to the liver, increased fatty acids synthesized within the liver, decreased oxidation of hepatic fatty acids, and impaired export of hepatic TAG as VLDL particles [20]. Accumulation of lipids within the hepatocyte is thus a reflection of the balance of these four key hepatic pathways, as well as proper regulation of the potential routes of fatty acid regulation within the hepatocyte. 

Hepatic uptake of NEFA is reflective of both the circulating NEFA concentration and blood flow to the liver, resulting in an increase in hepatic NEFA uptake during negative energy balance. During the periparturient period, both blood flow to the liver and the circulating NEFA concentration are increased and hepatic NEFA uptake can increase by thirteen times [15]. The degree of increased hepatic NEFA uptake is not matched with increased oxidative capacity which only minimally increases at calving compared to 28 days prior to calving [21,22]. Conversely, hepatic fatty acid esterification increases significantly at calving compared to precalving [21], highlighting the role of alternative pathways for hepatic fatty acid metabolism during the transition to lactation period.

The progression of ketosis and fatty liver is the response to poor adaptation to the challenges associated with the transition to lactation period. While the hepatic mechanism of bovine fatty liver and ketosis is not completely understood, it is clear that regulation of TAG uptake, lipolysis, oxidation, and storage play a key role in the onset and progression of the disorders.

## 3. Anaplerosis and Cataplerosis of the TCA Cycle

Maintaining a balance between anaplerosis, the synthesis of metabolic intermediates, and cataplerosis, the extraction of metabolic intermediates for breakdown, is critical in preserving hepatic carbon homeostasis. Anaplerosis was originally used to describe the flux of carbons into the tricarboxylic acid cycle (TCA) in *E. coli* [23]. Although anaplerosis is now used to describe additional biological reactions, the maintenance of TCA intermediates is still physiologically relevant [24] and may be key to understanding the etiology and potential intervention strategies in the onset of ketosis and fatty liver disease.

**Figure 1:**
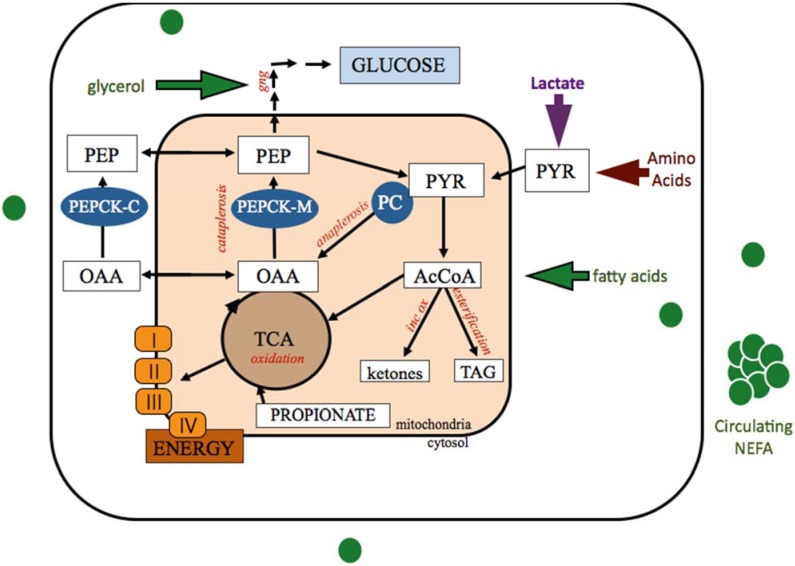
Anaplerotic and cataplerotic pull of oxaloacetate generated by pyruvate carboxylase carboxylation of pyruvate within the hepatocyte. Mobilized adipose stores provide NEFA and glycerol for hepatic uptake and metabolism. TCA cycle capacity is dictated by the OAA pool and determines how many AcCoA carbons can be completely oxidized. Alternatively, AcCoA carbons can be incompletely oxidized to produce ketone bodies or esterified into triglycerides for storage or export. Acetyl-CoA, AcCoA; cytosolic phosphoenolpyruvate carboxykinase, PEPCK-C; gluconeogenesis, GNG; incomplete oxidation (ketogenesis), inc ox; mitochondrial phosphoenolpyruvate carboxykinase, PEPCK-M; nonesterified fatty acids, NEFA; oxaloacetate, OAA; phosphoenolpyruvate, PEP; pyruvate carboxylase, PC; Tricarboxylic acid cycle, TCA; triglyceride, TAG.

In vertebrates, the TCA cycle functions to oxidize acetyl-CoA for energy production and to supply intermediates to other biological pathways such as gluconeogenesis and fatty acid synthesis (Figure 1). In ruminants, supply of carbons for gluconeogesis is primarily from propionate produced in the rumen, although lactate, amino acids (specifically L-alanine), and glycerol also provide carbons for glucose production [25]. Propionate enters the TCA cycle through succinate, thus providing carbons that can either remain within the TCA cycle, or can be extracted from the cycle for gluconeogenesis. Conversely, amino acids and lactate enter the TCA cycle through pyruvate conversion to either oxaloacetate (OAA) or acetylCoA [25]. When endogenous glucose demand exceeds propionate supply, the relative importance of these substrates is increased [25]. As thoroughly reviewed by Aschenbach (2010), the entry point of these substrates differs and is controlled by different isoforms of phosphoenolpyruvate carboxykinase (PEPCK), with the mitochondrial form (PEPCK-M) controlling entry of lactate and cytosolic form (PEPCK-C) controlling entry from amino acids and propionate [25].

The oxidative capacity of the TCA cycle is dependent on availability of OAA, which serves as the carbon carrier for the cycle. Supply of oxaloacetate originates from either the carboxylation of pyruvate by pyruvate carboxylase (PC) in mitochondria, or from net maintenance of propionate carbon within the TCA cycle (Figure 1). Oxaloacetate can either remain in the TCA cycle, continually regenerated, or can be decarboxylated and phosphorylated to phosphoenolpyruvate via phosphoenolpyruvate carboxykinase (PEPCK). This reaction allows carbons to enter gluconeogenesis and represents a cataplerotic pull of carbons away from the TCA cycle. In this way, carboxylation of pyruvate to OAA immediately increases the OAA pool size; however, carbons from propionate only provide additional OAA if they are not extracted from the TCA cycle into gluconeogenesis. 

Anaplerotic supply of OAA into the TCA cycle through increased PC activity serves to replenish or offset carbon loss due to cataplerosis. In the absence of cataplerotic pull, increased PC generation of OAA can increase the capacity of the TCA cycle, potentiating increased oxidation of acetyl-CoA. Therefore the role of PC in the formation of OAA, a pivotal molecule in metabolism, is critical to anaplerosis and cataplerosis in many tissues and impacts several biological pathways [24,26,27].

Cataplerosis is only possible if anaplerosis is adequate and the pool of TCA cycle intermediates is maintained [23,24,28]. Early research suggested that increased gluconeogenesis could deplete the OAA pool in the mitochondria, restricting complete oxidation of acetyl-CoA [29]. Since that time, animal studies have further supported this original hypothesis [3,19,22,30]; however, there is still limited research devoted to understanding the biochemical and molecular events that balance these pathways. The balance of cataplerosis and anaplerosis is specifically critical in the transition to lactation period in the dairy cattle life cycle, when both glucose and energy demands exceed availability. 

**Figure 2:**
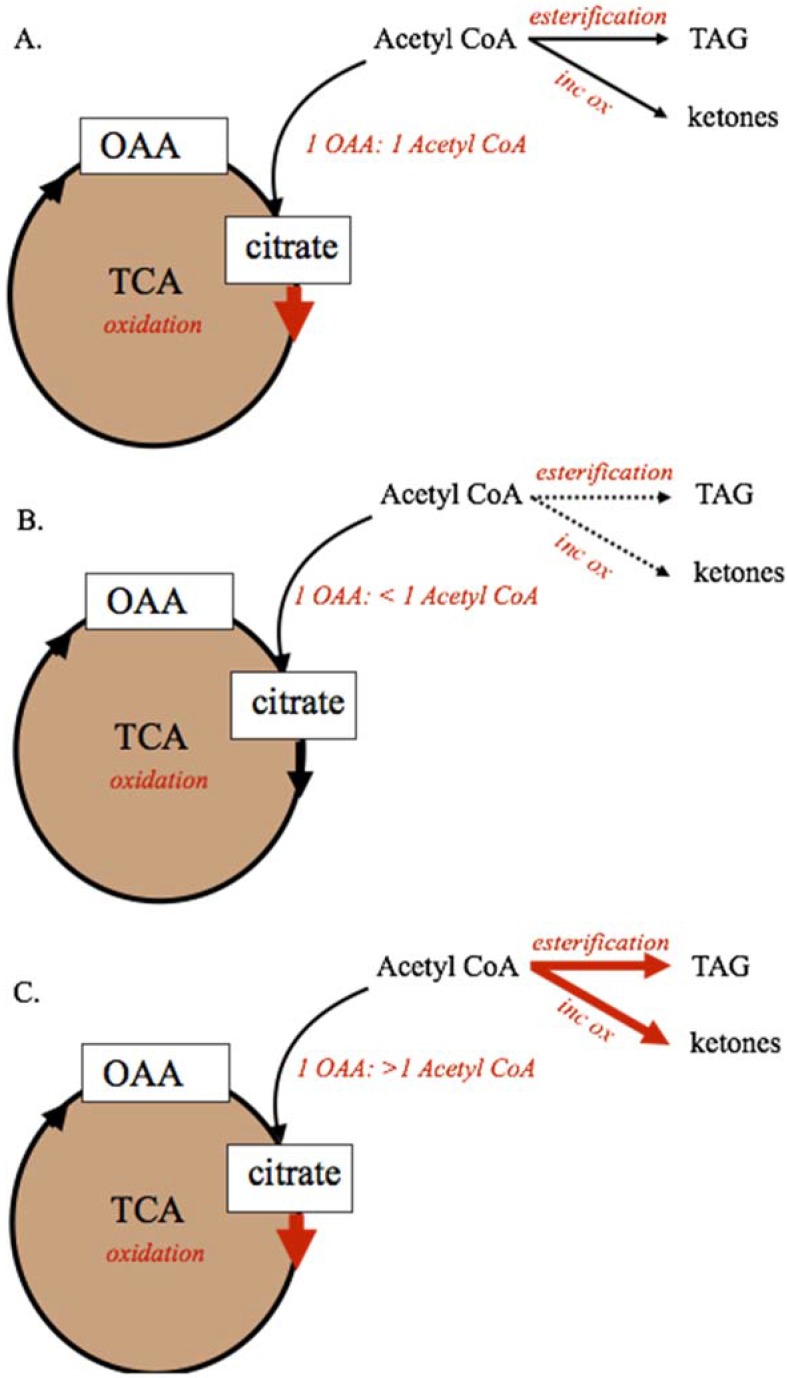
Mitochondrial metabolism of dietary and body reserve metabolites. For metabolic pathways shown, solid lines represent predominant pathways, dashed lines represent subsidiary pathways. The capacity of the tricarboxylic acid cycle to oxidize acetyl-CoA, and maintain anaplerosis, requires a 1:1 ratio of acetyl-CoA and oxaloacetate (Panel A). If 2-carbon precursor (acetyl-CoA) availability is not adequate to maintain the 1:1 ratio, complete oxidation by the TCA cycle will be decreased. If availability of 2-carbon precursors exceeds oxaloacetate availability (Panel C) either because aneplorosis is not maintained or because of excess acetyl-CoA supply, the TCA cycle capacity is exceeded and acetyl-CoA carbons must be alternatively metabolized through incomplete oxidation to ketone bodies or esterified as TAG for storage or export. Incomplete oxidation (ketogenesis), inc ox; oxaloacetate, OAA; tricarboxylic acid cycle, TCA; triacylglyceride, TAG.

During periods of feed restriction and adipose tissue mobilization, or when circulating NEFA and TAG are elevated due to diet or disease, the capacity of the TCA cycle to completely oxidize acetyl-CoA can be exceeded. As shown in Figure 2, the capacity of the TCA cycle to completely oxidize fatty acid-derived acetyl-CoA units is dependent on maintaining a 1:1 relationship between OAA and acetyl-CoA within the mitochondria [30,31,32,33]. Decreases in OAA supply that reduce this ratio to <1 OAA:1 Acetyl-CoA through decreased anaplerosis or increased cataplerosis, will decrease the capacity of the TCA cycle to oxidize acetyl-CoA. Conversely, if excessive supply of fatty acids to the liver increases acetyl-CoA supply beyond OAA availability, the TCA cycle capacity will be exceeded and metabolism of acetyl-CoA through ketogenesis or esterification to TAG could be increased. In these cases, ketogenesis of acetyl-CoA to acetate, acetoacetate, and BHBA, and esterification to TAG for storage are increased [3,22,30]. 

Examination of the balance of PC and PEPCK during different physiological states or during nutritional challenges highlights the importance of anaplerotic balance. Increased hepatic expression of PC, but not PEPCK, is observed at calving [34,35,36,37] and may allow for increased oxidative capacity within the mitochondria. Regulation of PC during the transition to lactation is through regulation of PC Promoter 1, which is upregulated by fatty acids that mimic those in circulation at calving [32,35,38,39]. Increased oxidative capacity at the time of calving is realized physiologically as demonstrated by increased total oxidation of palmitate in liver tissue slices [21]. Beta-oxidation of fatty acids within the peroxisome is a large contributor to this increased oxidative capacity and accounts for 50% of first cycle total palmitate β-oxidation in bovine liver [40]. Upregulation of gene expression for regulatory genes involved in these pathways have been observed and support patterns of increased oxidative capacity in both the mitochondria and peroxisome at calving [28,37,41]. Peroxisomal oxidation results in the initial release of hydrogen peroxide, rather than NAD, and unlike mitochondrial oxidation, is not linked to the respiratory chain for ATP production. Despite this lack of ATP production, peroxisomal β-oxidation plays a key role in partial oxidation of NEFA that exceed mitochondrial oxidative capacity [4]. Given that if not oxidized, the alternative metabolic fate of acetylCoA is ketogenesis or esterification; maintenance of the OAA pool for maximal TCA cycle capacity is critical to preventing excessive ketone body production and TG accumulation. 

The balance of PC and PEPCK can also serve to fuel cataplerosis and gluconeogenesis. When propionate supply from the rumen is increased post-calving, with rumensin, or during glycerol feeding, increased expression of PEPCK-C allows for cataplerosis of OAA carbons into gluconeogenesis for glucose production [25,42,43]. This shift would allow for increased gluconeogenic capacity when precursors are available and increased endogenous glucose production is required to support milk production [25,42,43].

Increased oxidative stress and inflammation during the peripurient period has been documented in dairy cows [28,37,41,44,45,46,47]. While the direct impact of oxidative stress and inflammation on TCA cycle capacity, mitochondrial efficiency, and hepatocyte function have not been specifically elucidated, it is likely that additional insults of inflammation and oxidative stress would further exacerbate the metabolic load associated with NEFA oxidation, ketosis, and fatty liver. In hepatic tissue from cows with induced-ketosis, genes involved in cytokine signaling, inflammation, oxidation (including PC), and esterification were upregulated compared with hepatic tissue from healthy cows [41]. Conversely, genes involved in gluconeogenesis, including PEPCK, were downregulated [41]. These coordinated changes allow for sustained fatty acid oxidation, ketogenesis, and esterification despite the onset of inflammation.

## 4. Conclusions and Future Directions

Pyruvate carboxylase catalyzes the generation of OAA from pyruvate, a critical anaplerotic and cataplerotic reaction that impacts TCA cycle capacity, gluconeogenesis, and fatty acid synthesis. Given that liver uptake of fatty acids and TAG is proportional to the concentration of fatty acids and TAG in circulation, understanding the limitations to hepatic fatty acid metabolism is critical to developing potential prevention or treatment strategies. Understanding the balance of anaplerotic and cataplerotic and the resulting shifts in the TCA cycle oxidation of acetyl-CoA, can provide biochemical insight into hepatic oxidative capacity and lipid accumulation. The balance of PC to PEPCK serves as a control point to shift net carbon flux towards glucose production when precursors are adequately available, or to favor TCA cycle oxidation and prevent OAA pool depletion when maximal oxidative capacity is required. Taken together, characterization of individual gene regulation and recent whole genome data, suggests that differential regulation of PC and PEPCK, and the resulting ratio, are a part of the coordinated response to optimize hepatic metabolism of the dynamic substrates available during the periparturient period. 
